# Glare-free retinal imaging using a portable light field fundus camera

**DOI:** 10.1364/BOE.9.003178

**Published:** 2018-06-20

**Authors:** Douglas W. Palmer, Thomas Coppin, Krishan Rana, Donald G. Dansereau, Marwan Suheimat, Michelle Maynard, David A. Atchison, Jonathan Roberts, Ross Crawford, Anjali Jaiprakash

**Affiliations:** 1 Queensland University of Technology, Brisbane, QLD 4000, Australia; 2 Medical and Healthcare Robotics, Australian Centre for Robotic Vision, Brisbane, QLD 4000, Australia; 3 Computational Imaging Lab, Stanford University, Stanford, CA 94305, USA; 4 Institute of Health and Biomedical Innovation, Brisbane, QLD 4059, Australia

**Keywords:** (230.0230) Optical devices, (170.4460) Ophthalmic optics and devices, (110.1758) Computational imaging, (100.6890) Three-dimensional image processing

## Abstract

We present the retinal plenoptoscope, a novel light field retinal imaging device designed to overcome many of the problems that limit the use of portable non-mydriatic fundus cameras, including image quality and lack of stereopsis. The design and prototype construction of this device is detailed and the ideal relationship between the eye pupil, system aperture stop and micro-image separation is investigated. A comparison of the theoretical entrance pupil size, multi-view baseline and depth resolution indicates that a higher degree of stereopsis is possible than with stereo fundus cameras. We also show that the effects of corneal backscatter on image quality can be removed through a novel method of glare identification and selective image rendering. This method is then extended to produce glare-free depth maps from densely estimated depth fields, creating representations of retinal topography from a single exposure. These methods are demonstrated on physical models and live human eyes using a prototype device based on a Lytro Illum consumer light field camera. The Retinal Plenoptoscope offers a viable, robust modality for non-mydriatic color and 3-D retinal imaging.

## 1. Introduction

Diseases of the retina affect millions of people worldwide. According to a 2012 World Health Organization study, age-related macular degeneration, diabetic retinopathy and glaucoma are estimated to be the cause of up to 5.4 million cases of blindness worldwide. A large number of these cases can be avoided if diagnosed early enough, monitored and treated appropriately [1].

The most common method of diagnosing diabetic retinopathy and age-related macular degeneration is by the use of mydriatic or non-mydriatic color digital fundus cameras. These are often bulky, fragile and expensive and the photographer must be technically trained. Much of the cost and complexity of modern fundus cameras is due to the sophisticated optical and mechanical assemblies needed to accurately align and focus them to the patient’s eye to provide even and glare free illumination to the retina.

Smaller handheld digital ophthalmoscopes and smartphone adapters have been developed to minimize costs and increase portability but these devices generally require an artificially dilated pupil and an experienced operator to take images [2]. Although there are many devices available in this class, they have failed to conform to the image quality standards required by major screening programs and have been excluded from use [3].

Most current fundus cameras use digital sensors to capture images, similar to a photographic DSLR camera which records an image as a 2-D light intensity map. Light field or plenoptic cameras differ in that they encode the intensity and direction of a ray at a sensor position. Microlens (also lenslet) based designs do this by multiplexing micro-images (images formed under a set of microlenses) on to the sensor, taken from different perspectives. 2-D images may then be reconstructed simulating novel viewing positions, different apertures and different focal planes by computationally taking an appropriate slice through the light field. Scene depth may also be calculated through feature or image based approaches [4].

Portable light field cameras have been commercially available since 2010 for photographic and industrial purposes. Their use for retinal imaging offers potential advantages over traditional fundus photography: post acquisition refocusing, glare reduction and the production of topographic depth maps. The ability to refocus an image as a post process reduces the necessity for the patient to properly focus their eyes during acquisition. Corneal glare may be identified in the light field and avoided during reconstruction of 2-D images and 3-D depth maps. These features can reduce the complexity of the imaging and illumination optical systems and hence the size and cost of the instruments. Finally, the parallax developed between views in light field imaging could be exploited to create topographical maps of the retina for improved diagnosis.

The aims of this study are to:

- Design, build and test the first non-mydriatic light field fundus camera. We refer to this new instrument as a Retinal Plenoptoscope, an ‘all seeing examiner’ for the eye.
- Demonstrate post-acquisition removal of corneal glare from retinal images.
- Demonstrate the creation of topographical depth maps of the human retinal fundus from a single exposure.

## 2. Background

Color fundus photography is the gold standard for the detection and monitoring of retinal diseases. Most fundus camera designs feature a well corrected objective lens, relay optics and a sensor for recording the image [5]. They may also have a fixation target for directing the patient’s gaze and a flash system to evenly illuminate the imaged part of the retina, typically providing fields of 30–45°. Non-mydriatic versions can be successfully used through pupils as small as 4 mm [6–8].

Glare is an angularly dependent optical phenomena caused from the reflectance of flash illumination from the interface between media of differing refractive index. There are four main reflections in the eye (the Purkinje images), involving the anterior and posterior surfaces of the cornea and the anterior and posterior surfaces of the lens. The most detrimental of these to retinal imaging is that caused by the tear film and anterior corneal surface. The corneal reflection (or glare) has a higher intensity than the fundus because the latter has a low reflectance [9]. This problem is often overcome by separating illuminating and imaging optical paths, with the imaging path being centered in the pupil and the illumination being provided in a toroid around it [10].

A sense of the relative depth of retinal structures, particularly of the optic nerve and macula is used to increase accuracy of diagnosis in cases of glaucoma and macular edema [11]. Stereo fundus photography has traditionally been used to support this need. Stereo retinal images may be acquired by taking multiple monocular images at various poses (sequential) or by splitting the imaging path inside the camera and viewing through two separate entrance pupils (simultaneous). The degree of stereopsis is determined by the center distance between the two entrance pupils (stereo or multi-view baseline) as seen at the pupil plane of the eye. As geometric separation between imaging and illumination must also be maintained, artificial dilation of the pupil may be required to achieve sufficient stereopsis to accurately measure depth.

A photogrammetric method to determine the curvature of the retina was proposed using structure from motion and an affine camera model [12]. This technique used retinal tree feature locations extracted from pairs of 2-D images and fitted them to a spherical model via a constrained bundle adjustment optimization. A similar method using multi scale gradient detection of the retinal tree was demonstrated using multiple wide angle 2-D images taken at different viewpoints [13]. A method of 3-D reconstruction of the optic disk from stereo pairs of fundus images has also been demonstrated [14], with correction for defocus and radiometric differences between images, rectifying the images for pose and matching features to solve a global optimization problem. These methods require multiple images to be taken of sufficient quality and similarity to perform robust feature detection and registration, which is difficult to achieve using portable non-mydriatic cameras.

Light field imaging combines recent advances in computing power and sensor technology with theory developed from the biological science [15] and computer graphics communities [16]. Light field cameras record the light transport in a scene in terms of geometrical optics, with each light ray being described as a 4-D vector with two directional terms and two spatial terms. Computational manipulation of the captured light field allows photographers to synthetically change the focus and aperture setting of images after acquisition, acquire depth estimates [17] and identify internal camera glare in extended scenes [18]. Light field rendering is a process for creating 2-D images from the light field and can be done from various perspectives, synthetic apertures and focal depths using existing techniques [17, 19, 20]. These techniques are image based, applied universally to the light field and are non-selective of scene content.

In the biological imaging domain, light field imaging has been applied to microscopy [21], otoendoscopy [22] and endoscopy [23]. There have been foundational studies and simulations performed on the theoretical viability of light field retinal imaging [24] and its potential to determine depth through tissue [25]. These studies concluded that light field retinal imaging would be a feasible and attractive proposition but did not discuss the methodology for capturing or processing such light fields. To the best of our knowledge there is only one published academic work discussing the use of a practical system [26]. In this case a commercial light field camera was used to perform mydriatic indirect ophthalmoscopy through a handheld objective lens and demonstrate post acquisition refocus of clinical images. However, the system was not optimized for retinal imaging and required a skilled practitioner to operate. We extend this idea to non-mydriatic fundus photography by designing and testing a light field fundus camera with integrated eye fixation, illumination and imaging systems. We also develop image processing techniques to remove corneal glare and produce depth maps of the retinal fundus.

## 3. Methods

### 3.1. Design considerations

**Fig. 1:**
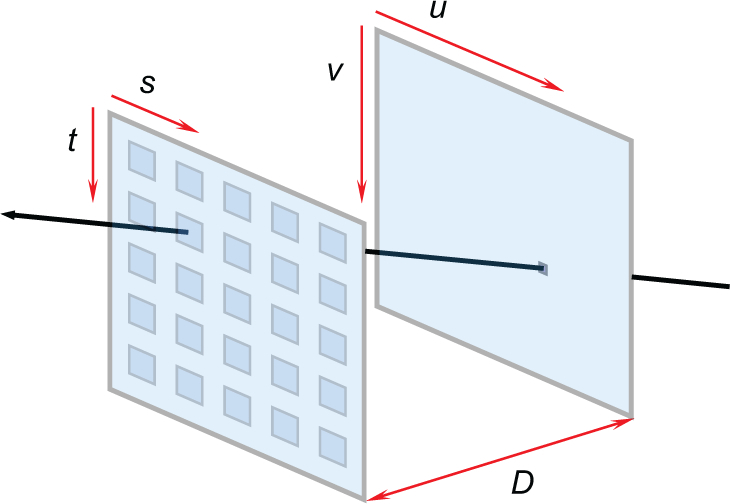
Two plane parameterization of light rays - The light field can be defined as a four dimensional array *L*(*u, v, s, t*) where each ray is categorized by the points at which it intersects two arbitrary parallel planes (*u, v*) and (*s, t*) separated by an arbitrary distance *D*.

There are two variants of microlens based light field sensors in the literature with the unfocused type being the basis of our system. We will define the light field in terms of its two plane parameterization (2PP) as *L*(*u, v, s, t*), depicted in Fig. 1 below.

**Fig. 2:**
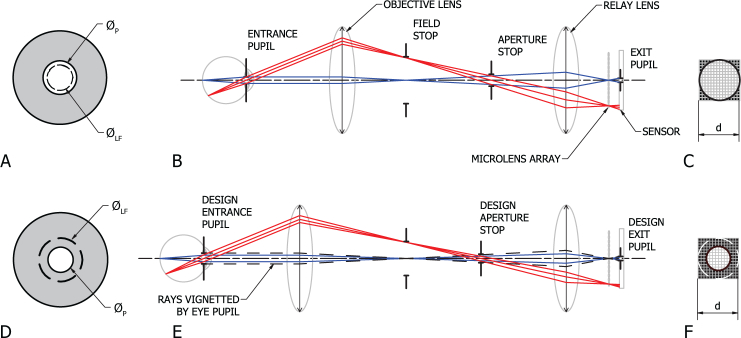
Imaging path optical diagram of light field fundus camera. Top row (A,B,C) represents a correctly designed system where the entrance pupil diameter Ø*_LF_* is smaller than the eye pupil Ø*_P_* and the region of the sensor under the microlens shows minimal vignetting, where *d* is the number of pixels under a microlens horizontally and vertically. Bottom row (D,E,F) represents an incorrectly designed system where Ø*_LF_* is larger than Ø*_P_*. The resultant micro image vignetting is shown in (F).(A) and (D) show slices taken approximately through the iris of the eye. (B) and (D) show the arrangements of components and paraxial approximations of the ray paths for a point on (blue) and off-axis (red). The design entrance and exit pupils are the images of the design aperture stop as seen through the objective and relay lenses respectively.

In a light field camera the images formed under the microlenses are separated on the sensor by sizing and positioning the system exit pupils such that they do not blur together, but are also not severely vignetted. This is done by matching the focal length / aperture ratio (f-number) of the microlenses to that of the image side of the main lens system, resulting in a system of fixed aperture ratio [27]. For a fixed focal length photographic camera, this is simply a matter of designing the correct aperture stop for the system. Non-mydriatic retinal imaging, however, occurs through the eye’s entrance pupil which can change diameter through a range of 2–8 mm depending on external lighting conditions and the condition and age of the patient. As this is a large variation, we must determine the minimum ocular pupil size Ø*_P_* we wish to view through. We must then ensure that the limiting aperture in the system is always part of the camera and produces optimum microlens coverage on the sensor while eliminating cross talk. Fig. 2(B) shows a simplified imaging optical path for a light field fundus camera and the preferred arrangement of pupils and stops. If the physical aperture in the camera is too large, the aperture stop becomes the eye pupil and the micro images become vignetted, limiting the number of angular samples of the lightfield as shown in Fig. 2(F). A smaller aperture stop will not create cross talk but will reduce the amount of light received to the sensor and decrease the multi-view baseline available for depth estimation.

**Fig. 3:**
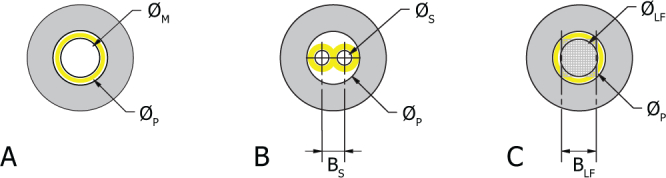
Entrance pupils, stereo (*B_S_*) and multi-view (*B_LF_*) baselines for arbitrary eye pupil diameter Ø*_P_*; (A) Mono fundus camera (Ø*_M_*), (B) stereo fundus camera (Ø*_S_*), (C) Light field fundus camera (Ø*_LF_*). Illumination paths shown in yellow. Grid on (C) represents light field sampling pattern at entrance pupil.

The entrance pupil in a fundus camera must be smaller than the pupil of the eye and is further limited in size to reduce aberrations and allow for geometric separation of the illumination and imaging paths as shown in Fig. 3(A). An entrance pupil diameter Ø*_M_* of less than 2 mm is recommended [9] for a monocular fundus camera. A typical stereo fundus camera entrance pupil arrangement is shown for comparison in Fig. 3(B). The entrance pupil diameter Ø*_S_* is calculated by Eq. (1) assuming equal pupil areas for imaging and illumination [28] and optimal filling of the eye pupil:

$$\emptyset_{S}=\frac{2\emptyset_{P}}{3\sqrt{2}+1}$$ (1)

The effective stereo baseline, *B_S_*, of this system is determined by Eq. (2):

$$B_{s}=\frac{(\sqrt{2}+1)\emptyset_{S}}{2}$$ (2)

In a light field fundus camera, the entrance pupil diameter Ø*_LF_* is equivalent to that of a standard monocular fundus camera Ø*_M_* and can be calculated by Eq. (3):

$$\emptyset_{LF}=\sqrt{\frac{\emptyset_{P}^{2}}{2}}$$ (3)

In this case, the total multi-view baseline *B_LF_* of the system is related to the entrance pupil diameter Ø*_LF_* by Eq. (4), where *d* is the number of pixels per microlens as illustrated in Fig. 2(C):

$$B_{LF}=\frac{\emptyset_{LF}(d-1)}{d}$$ (4)

The effect of stereo baseline on depth resolution is given in [5] and is reproduced as Eq. (5):

$$Z_{min}=\frac{4{\left(f_{eye}^{\prime }\right)}^{2}\lambda }{B_{S}\emptyset_{S}}$$ (5)

where $f_{eye}^{\prime }$ is the image side focal length of the eye and *λ* is the wavelength of light used, 550nm is used for calculations in this paper. *B_LF_* and Ø*_LF_* may be substituted into the denominator of Eq. (5) for comparison with a light field fundus camera.

The angular sampling rate of an ideal unfocused light field camera is equal to the number of pixels (*d*) under each microlens in each extent of the plane parameters *s* & *t*. The microlens diameter, and by extension, the exit pupil size will be determined by the pixel size of the sensor and the desired angular sampling rate. The entrance and exit pupil sizes for the camera are therefore fixed in size, the former by the diameter of the non-mydriatic pupil of the eye and the latter by the choice of microlens array size and sensor arrangement. This constrains the objective and relay lens combination and should be considered by the designer of these systems in conjunction with design parameters such as desired field of view and working distance.

### 3.2. Optical design

The optics for the prototype Retinal Plenoptoscope were designed using paraxial and thin lens approximations and 2-D geometric ray tracing implemented in MATLAB. The chief design constraint was that all parts must be easily available, including the light field sensor which is not available as parts and can only be purchased as part of a camera. To check the relationships between the entrance pupil diameter Ø*_LF_* and effective focal length (EFL), we modeled the system in Zemax OpticStudio using library data for the objective and relay lenses. The optics of the Lytro Illum camera we selected are unknown so we model this system as a thin lens with the focal length retrieved from image properties exported from the camera. Aberrations of the eye and lens system were ignored for this work.

### 3.3. Imaging procedure

To setup and test camera imaging and illumination optics for extended durations and determine the field of view and spatial resolution of the imaging system, we used the Epipole physical model eye [29]. Its optics are based on the Le Grand schematic eye and it has a 4 mm entrance pupil. Its chambers are filled with demineralized water and the retinal plane is fitted with a negative USAF 1951 resolution target. Resolution tests were performed with the model eye backlit through the target using a white LED light source.

Following ethics approval from The Queensland University of Technology’s human research ethics committee, the Retinal Plenoptoscope was tested on consenting participants. Each eye was examined by a qualified optometrist for refractive error. The participant’s eye was imaged in a minimally lit room without artificially dilating the pupil.

**Fig. 4:**
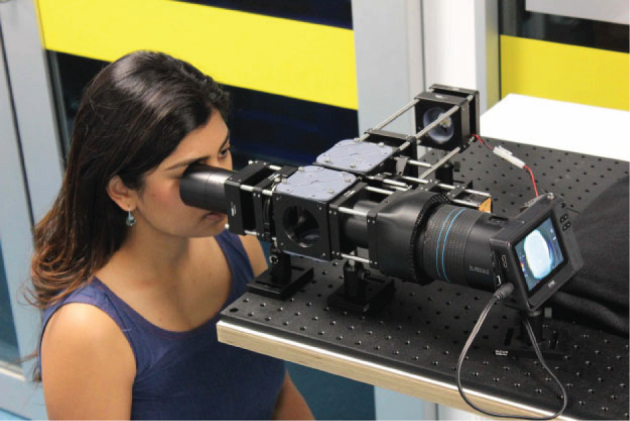
Retinal Plenoptoscope in use.

No head restraint was used. Fixation and alignment were reliant on the patient interaction with the fixation target alone. A forehead rest was provided to help maintain a nominal working distance, but this constraint was not rigorously enforced. The operator had no visual feedback through the imaging process, the only controls being ON/OFF and FIRE. Figure 4 shows the prototype setup.

### 3.4. Preprocessing & decoding

The raw Lytro .LFR image captured by the sensor is a hexagonal array of circularly vignetted micro-images. The light field was extracted from this data in a decoding process, using the functions available in the LF Toolbox V0.4 [30], to produce a debayered regular 4-D light field structure in the two plane parameterized (2PP) form.

A light field may be expressed as a set of 4-D coordinates **D** and a mapping *L* that prescribes a value in the red-green-blue (RGB) color space for every coordinate in **D**. The set **D** is defined in Eq. (6) as:

$$D≔\{(u,v,s,t)\in Z^{4}|u_{min}\leq u\leq u_{max}\wedge v_{min}\leq v\leq v_{max}\wedge s_{min}\leq s\leq s_{max}\wedge t_{min}\leq t\leq t_{max}\}$$ (6)

The mapping *L* is defined as *L*: **R**^4^ ⇒ [0, 1]^3^, where *L* is well defined over the set **D** and the image of **D** under *L* is **L**. Thus *L* maps the set **D** to the set **L** as shown in Eq. (7):

L:D⇒L (7)

### 3.5. Glare identification

As corneal glare is a specular reflection, we assume that the backscatter will be of the same spectrum as the flash. We also assume that the glare will have a much higher intensity than the retinal image. Thus we can identify glare in the light field by finding regions of both high intensity and low saturation.

We can express glare as the set **G**, which is the set of all pixels for which the intensity *f* is higher than a threshold *C*_1_, and the saturation *S* is lower than a threshold *C*_2_, as given by Eq. (8):

$$G≔\{(r,g,b)\in {\left[0,1\right]}^{3}|f(r,g,b)\geq C_{1}\wedge S(r,g,b)\leq C_{2}\}$$ (8)

The set of glare in a light field, **B** ⊂ [0, 1]^3^, is then given in Eq. (9) by the set of points that exist in both the set of glare points **G** and the set of RGB light field points **L**:

B≔G∩L. (9)

The location of glare in a light field, **K** ⊂ **Z**^4^, may now be found by inverting the mapping *L* such that **K** is the preimage of **B** under *L* as shown in Eq. (10):

L:K⇒B (10)

We can now define a glare mask for the light field, *M*, that is 0 for all points that correspond to glare and 1 otherwise, as:

$$M(u,v,s,t)=\{\begin{array}{cc} 0 & (u,v,s,t)\in K\\ 1 & (u,v,s,t)\notin K \end{array}$$ (11)

### 3.6. Glare-free rendering

Glare in the light field behaves similarly to an occlusion, and given each scene point is sampled by multiple rays, it is possible to reconstruct 2-D images by using information from views not occluded by glare. To do this, we extend basic light field rendering techniques to allow for glare masking and blending, using a method similar to [31]. Glare masking refers to ignoring parts of the light field that correspond to glare, and blending refers to integrating a single point from a range of different views in the light field. This means that a rendered image is generated by choosing the focal plane at the fundus, and ‘looking around’ the glare objects in the scene to see the retina behind them.

The glare-free render may be expressed as an equation of the form:

$$p(i,j)=\frac{\sum_{k=\frac{n-1}{2}}^{\frac{n-1}{2}}\sum_{l=\frac{n-1}{2}}^{\frac{n-1}{2}}\left(L(i+k,j+l,\frac{d}{2}+mk,\frac{d}{2}+ml)\times M(i+k,j+l,\frac{d}{2}+mk,\frac{d}{2}+ml)\right)}{\sum_{k=\frac{n-1}{2}}^{\frac{n-1}{2}}\sum_{l=\frac{n-1}{2}}^{\frac{n-1}{2}}M(i+k,j+l,\frac{d}{2}+mk,\frac{d}{2}+ml)}$$ (12)

where *p*(*i, j*) is a point in the output image, *L* and *M* are mappings defined above, *d* is the size of the microlens, *m* refers to focal depth of the render, and *n* refers to the number of views over which integration occurs.

### 3.7. Depth mapping

We can calculate the corresponding depth field for the light field using a standard method employing gradients [32]. The depth field created this way can be thought of as a set of coordinates **D**, as defined above, and a mapping *P_Z_*: **Z**^4^ ⇒ **R** that prescribes a relative depth value for every point in **D**. The mapping is further defined in Eq. (13) as:

$$P_{Z}=\frac{D}{1+\frac{\partial L(n)/\partial s}{\partial L(n)/\partial u}}$$ (13)

Where *D* is the plane separation defined by the 2PP and *L*(*n*) is the intensity of the light field for one of the RGB color channels.

We can create a depth map that provides a 2-D representation of depth in the light field by performing a glare-free render of the depth field. This is done by substituting the mapping *P_Z_* for the mapping *L* in Eq. (12) above.

## 4. Results

### 4.1. Design of prototype equipment

**Fig. 5:**
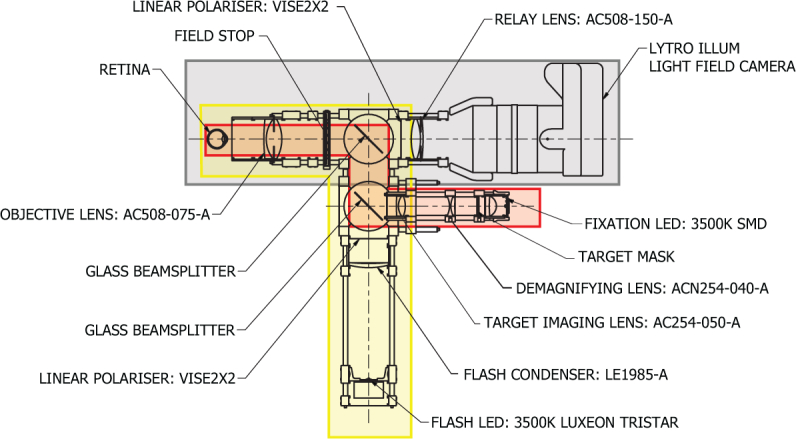
Plenoptoscope - General arrangement. Imaging path in gray, eye fixation path in red, illumination path in yellow. The Lytro Illum light field camera has an internal fixed f/2.0 aperture stop (not shown).

The prototype light field fundus camera was developed around a Lytro Illum consumer light field camera, which has an unfocused or ‘Plenoptic 1.0’ type microlens-sensor configuration at a constant f/2.0. The camera was mounted on a custom adapter to align the optical axis to that of the plenoptoscope. The shutter was synchronized with the flash via a microcontroller and a transistor switching board. The remainder of the plenoptoscope can be divided into optical subsystems for imaging, eye fixation and illumination (Fig. 5).

### 4.2. Imaging system

We used a 50.8 mm diameter, 75 mm focal length achromatic doublet as the objective lens. This design offers good correction for spherical aberrations and excellent chromatic reproduction. A second achromatic doublet is used as a relay lens. Internal backscatter from the interfaces of the objective lens is reduced through cross polarization. An adjustable field stop is positioned at the retinal conjugate plane on the sensor side of the objective. The system focal length can be easily changed using the zoom lens on the camera. The calculated effective focal lengths and entrance pupil diameters at various zoom settings are shown in Table 1.

**Table 1:** Microlens vignetting due to change of entrance pupil diameter for a fixed f/2.0 light field fundus camera. Effective focal length and entrance pupil diameter calculated from Zemax model, angular resolution from test images.

Lytro zoom setting (mm)	70	100	150
Effective focal length (mm)	−7.1	−9.4	−12.8
Entrance Pupil Diameter (mm)	3.5	4.7	6.4
Angular resolution (samples)	15	12	7

### 4.3. Eye fixation system

A target helps direct gaze and control the patient’s eye’s accommodation, ideally fixed at optical infinity. We have used an LED backlit fixation target at a retinal conjugate plane. Fixation on the central target position produces a field centered on the fovea; other fields may be acquired by viewing targets at various offsets. The target image is viewed by the imaged eye through two glass beam splitters and imaging optics as shown in Fig. 5.

### 4.4. Illumination system

We used an LED source in a similar arrangement to that of Suheimat [33]. As the light is delivered to the eye in three off axis spots focused at the corneal surface on a 3.5 mm pitch circle diameter, corneal glare is moved away from the center of the pupil. Plain glass beam splitters are used to direct the flash illumination into the objective lens. To avoid damage to the eye, the flash conforms to maximum radiant exposure guidelines [34] for photochemical, photoacoustic and thermal damage according to Standards Australia AS/NZS IEC 60825.1:2014 - Safety of laser products (Part 1: Equipment classification and requirements).

### 4.5. Optomechanical design

All optical components were mounted in a 60 mm cage system allowing for adjustment in the longitudinal axes while restraining translations and rotations.

### 4.6. System validation

**Fig. 6:**
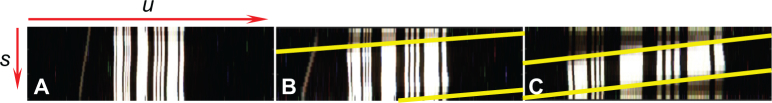
Epipolar slices (*s*-*u* plane) of model eye at EFL of (A): −7.1,(B): −9.4 and (C): −12.8 mm. Yellow lines show vignetting by 4 mm pupil.

Images were taken of the backlit model eye at increasing focal lengths while maintaining a fixed f/2.0 aperture to demonstrate the effects of f-number mismatch. The *s*-*u* epipolar diagrams in Fig. 6 are horizontal slices through the light field; shortening of the lines indicates the extent of vignetting caused by the 4 mm pupil of the model eye as it becomes the limiting system aperture. Unevenness top to bottom is caused by horizontal misalignment of the eye to the optical axis of the camera. It is notable that the edge of the pupil forms a diagonal stripe, this is the epipolar gradient at which the pupil exists and is equivalent to its relative depth in scene.

Using a captured light field of the model eye containing the USAF 1951 resolution target, the highest spatial resolution was found to be 17.96 lp/mm for a rendered in focus 2-D image at a focal length of −7.1 mm. A section through a line pair with normalized contrast > 0.5 was considered resolvable. The field of view was determined to be approximately 32 degrees.

**Fig. 7:**
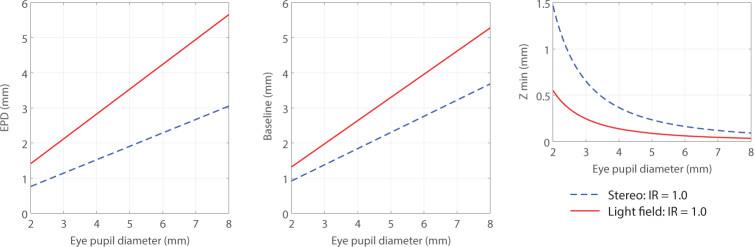
Comparison of calculated entrance pupil diameter, multi-view baseline and minimum resolvable depth (*Z_min_*) for a range of eye pupil diameters. A toroidal illumination system with an Illumination Ratio (IR) of 1.0 was assumed for both stereo and light field fundus cameras.

The multi-view baseline of the system was calculated for a range of iris sizes. Figure 7 shows the entrance pupil diameter, baseline and minimum resolvable depth (*Z_min_*) as calculated using Eq. (5) between systems B and C from Fig. 3.

### 4.7. Refocussing images and depth mapping

**Fig. 8:**
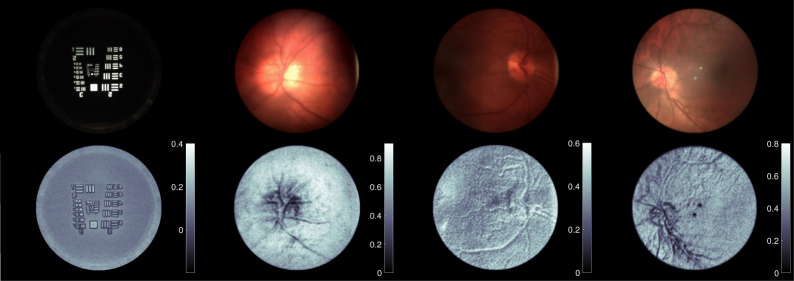
Series of images captured using the Retinal Plenoptoscope. Images are shown in sets of two with the top image being a standard (not glare-free) render of the light field, and the bottom image being a gray-scale relative depth map. Each depth map has an associated scale that relates gray shade to depth as defined by Eq. (13). Note that the leftmost set is of a model eye, the second leftmost set is of a myopic eye (−5.75D), and the two rightmost sets are of emmetropic eyes.

**Fig. 9:**
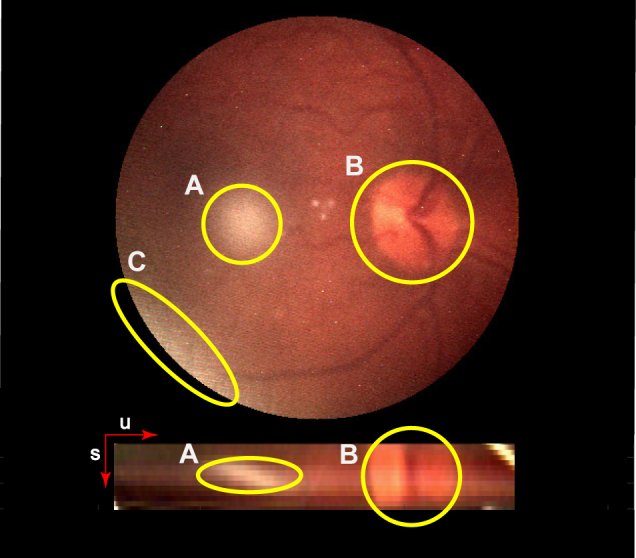
An image of a human retina captured using the Retinal Plenoptoscope with an associated epipolar image. Annotations indicate areas of interest, where (A) and (C) correspond to glare, and (B) corresponds to the Optic Disk.

Figure 8 shows the results of various captured light fields and depth fields refocused so that the retinal plane is in focus. Important features in the images are shown using an example in Fig. 9.

### 4.8. Glare reduction

**Fig. 10:**
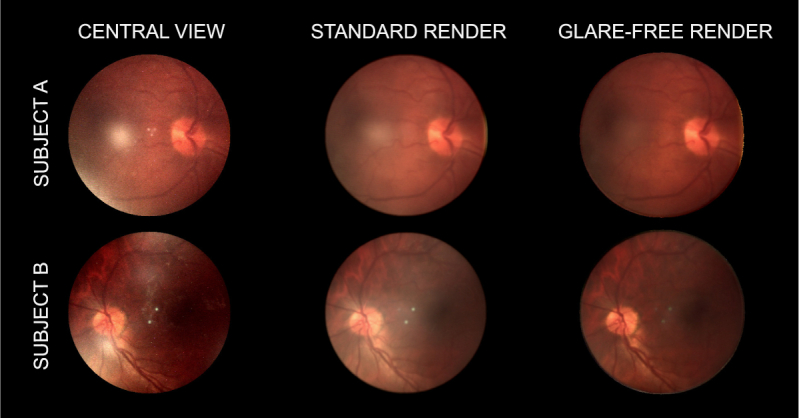
Series of images created using various light field rendering techniques. Images are shown in sets of three with the left image being the central view from the light field, the middle image being a standard render with no glare masking, and the right image being a glare-free render.

**Fig. 11:**
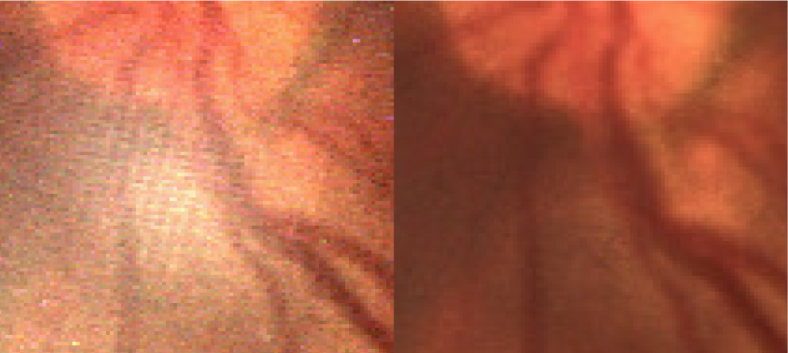
A zoomed-in section of a image of a human retina. The left image is the central view of the light field, and the right image is the glare-free render.

Figure 10 shows the result of using various different light field rendering techniques, including the glare-free render. Fig. 11 shows a zoomed-in section of one of the images.

## 5. Discussion

We have designed and built a new type of retinal imaging system which we have named the Retinal Plenoptoscope. We have demonstrated the ability to remove undesirable reflections from images and create depth maps from a single shot.

The diagnosis of retinal diseases such as macula edema, diabetic retinopathy and glaucoma are better performed with a sense of depth. Simultaneous stereo fundus photography suffers from a shortage of stereo baseline when performed through non-dilated pupils and sequential stereo fundus photography is not possible without accurate alignment of the eye that is not usually possible with portable equipment. Light field fundus photography offers a physically simple and robust 3-D imaging platform that also presents new solutions to the problems of focusing and corneal glare encountered with portable non-mydriatic retinal imaging.

The design of microlens based light field fundus cameras is governed by the design of the limiting system aperture. Results in Fig. 7 and Table 1 show the effects of incorrect pupil size or f-number mismatch on effective aperture and thus multi-view baseline. Our microlens based Retinal Plenoptoscope design, using a single imaging path, can utilize a larger portion of the available ocular exit pupil to achieve greater multi-view baseline than existing stereo fundus cameras. In addition, the geometric separation between imaging and illumination paths may be reduced as the removal of corneal glare can be performed as a computational post-process. Thus larger entrance pupil sizes may be used for imaging when compared with equivalent stereo fundus cameras. A further study into the optimization of light field fundus cameras including optic, plenoptic sampling and algorithmic design is necessary to fully understand the parameters to ensure optimal sampling ratios, optimum illumination ratios and aberration minimization.

In the retinal images and constructed relative depth maps in Figs. 8–11, the optic disk and retinal tree are clearly apparent. These features are visible as they are areas of high contrast on the retina, which is generally a low contrast environment. As noted by Deguchi [35], the virtual retinal image is approximately quadratic and flattened compared to the true topography. The depth maps in Fig. 8 show this, as the majority of the retina appears at a similar depth, with significant variations only occurring at the visible features including the retinal tree and the optic disk. A method to correct for this distortion has been implemented in optical coherence tomography applications and a light field solution to this problem should be investigated [36]. A limitation of the depth estimation technique used is that it relies on intensity change to measure depth, and so is biased towards areas of high contrast such as the retinal features. This limitation is not unique to retinal imaging, and is common to most forms of image and feature based depth calculation including light field and stereo.

The optics of the eye also provide a variable magnification depending on the working distance and state of accommodation at the time of image acquisition. The refractive elements of the eye, the cornea and crystalline lens, also produce unmodeled distortions and aberrations that we ignore in this paper but will affect the accuracy of the depth estimation. A calibration method that can accurately determine the intrinsic and distortion parameters of the entire imaging system including the refractive elements of the imaged eye at the moment of capture would address this issue and as such, is desirable and is left as further work.

Fig. 10 illustrates that the standard render is able to remove some of the glare from the image, but leaves behind a soft glow. This is due to the glare being integrated into the surrounding region and the overall ‘whiteness’ of the area being incorrectly increased. The glare-free render is able to successfully remove more of the glare, and does not introduce this ‘glow’ into the image, showing that it is superior to a standard render. The glare-free render is not able to remove all of the artifacts however, most visibly being the reflex from the glass-glass interfaces of the achromatic doublet used as an objective lens. This is still visible in the central region of the bottom-rightmost image of Fig. 10 due to the thresholds used not being tuned correctly for this type of glare artifact. For the case where glare exists across all views of the image, the affected region would appear black as the visual information could not be recovered. It is also clear that both rendering methods are able to recover large portions of the image that were previously obscured.

**Fig. 12:**
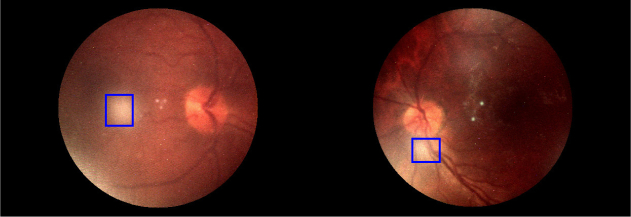
Diagram showing the ROI used to calculate the CNR for each image in Fig. 10. The CNRs calculated for the left image were: central view = 15.3, standard render = 10.2, glare-free render = 6.9. The CNRs calculated for the right image were: central view = 22.1, standard render = 14.4, glare-free render = 10.8.

To further analyze the effect of using the glare-free render we can examine the quality of images produced using the different rendering techniques. The peak signal-to-noise ratio (PSNR) and structurally similar (SSIM) metrics are not appropriate for these images as we have no similar noise-free images to compare with. Instead, we use the contrast-to-noise ratio (CNR), which measures the similarity of a region of interest (ROI) to the image background, and choose a visually identified area of glare as the region of interest (Fig. 12). Note that the optic disk was excluded from the background due to being an area of high contrast.

The CNR of the glare-free renders is less than that of the standard render, which is in turn less than that of the central view. This is further evidence that the glare-free render is superior to the standard rendering technique and that both are able to improve the quality of captured images. The reason for this is that the ‘background’ of the image (the retina) is generally a low-contrast environment. The main areas of high contrast are glare, the retinal tree to a lesser extent, and the optic disk. Thus a high CNR ratio for the chosen ROIs indicates a poor quality image. In both cases, the CNR of the ROI was approximately halved (from the central view to the glare-free render), which indicates a large increase in image quality due to a decrease in glare. Note that the CNR of the second image set is higher due to the inclusion of a part of the retinal tree in the ROI.

The ability to detect ray angles by signal multiplexing comes at the cost of spatial resolution. The Lytro Illum has a sensor with in excess of 40 million pixels, yet delivers images at an output resolution of 635 × 433 pixels. This decreases considerably the achievable resolution in comparison with a well designed monocular fundus camera with similar field of view. DeHoog et al. set a design MTF of > 0.40 at 50 lp/mm for their test cases, significantly higher than the 0.50 at 18 lp/mm shown in this study [10]. There are solutions to this problem using a different sensor arrangement that provides a better trade-off between spatial and angular resolution [37]. Coupled with super resolution techniques [38, 39], a state of the art light field fundus camera should be able to provide similar resolution to current alternatives. Nevertheless, it could be clinically useful to trade some spatial resolution for angular resolution to enable depth sensing, resilience to glare and post-acquisition refocusing. These features will improve the performance of the low cost, portable and handheld fundus cameras that will be required to bring regular and reliable quality retinal imaging to remote and rural areas where they are required the most.

### 6. Future work

Further studies are needed to improve the spatial resolution and image quality of the system to ensure it is suitable for screening programs and research databases. Also, clinical studies and comparison with existing imaging modalities, particularly color and stereo fundus cameras are required. Further work is also needed to develop a robust method of calibration and reconstruction that includes the imaging parameters of the system at the time of acquisition. This will allow metric measurements to be made of the three dimensional topography of the retina.
